# Atypical instantaneous spatio-temporal patterns of neural dynamics in Alzheimer’s disease

**DOI:** 10.1038/s41598-023-50265-3

**Published:** 2024-01-02

**Authors:** Sou Nobukawa, Takashi Ikeda, Mitsuru Kikuchi, Tetsuya Takahashi

**Affiliations:** 1 Department of Computer Science, Chiba Institute of Technology, 2-17-1 Tsudanuma, Narashino, 275-0016 Chiba Japan; 2 Research Center for Mathematical Engineering, Chiba Institute of Technology, 2-17-1 Tsudanuma, Narashino, 275-0016 Chiba Japan; 3 Department of Preventive Intervention for Psychiatric Disorders, National Institute of Mental Health, National Center of Neurology and Psychiatry, 4-1-1 Ogawa-Higashi, Kodaira, 187-8661 Tokyo Japan; 4 Research Center for Child Mental Development, Kanazawa University, 13-1 Takaramachi, Kanazawa, 920-8640 Ishikawa Japan; 5 United Graduate School of Child Development, Osaka University, Kanazawa University, Hamamatsu University School of Medicine, Chiba University, and University of Fukui, 2-2 Yamadaoka, Suita, 565-0871 Osaka Japan; 6 Department of Psychiatry and Behavioral Science, Kanazawa University, 13-1 Takaramachi, Kanazawa, 920-8640 Ishikawa Japan; 7 Department of Neuropsychiatry, University of Fukui, 23-3 Matsuoka, Yoshida, 910-1193 Fukui Japan; 8 Uozu Shinkei Sanatorium, 1784-1 Eguchi, Uozu, 937-0017 Toyama Japan

**Keywords:** Neuroscience, Diseases

## Abstract

Cognitive functions produced by large-scale neural integrations are the most representative ‘emergence phenomena’ in complex systems. A novel approach focusing on the instantaneous phase difference of brain oscillations across brain regions has succeeded in detecting moment-to-moment dynamic functional connectivity. However, it is restricted to pairwise observations of two brain regions, contrary to large-scale spatial neural integration in the whole-brain. In this study, we introduce a microstate analysis to capture whole-brain instantaneous phase distributions instead of pairwise differences. Upon applying this method to electroencephalography signals of Alzheimer’s disease (AD), which is characterised by progressive cognitive decline, the AD-specific state transition among the four states defined as the leading phase location due to the loss of brain regional interactions could be promptly characterised. In conclusion, our synthetic analysis approach, focusing on the microstate and instantaneous phase, enables the capture of the instantaneous spatiotemporal neural dynamics of brain activity and characterises its pathological conditions.

## Introduction

Cognitive functions are the most representative ‘emerging phenomenon’ in large-scale neural integration systems (reviewed in^1–3^). Recent advances in brain network science have provided powerful insights into a wide range of spatiotemporal neural integrations underlying various cognitive functions (reviewed in^4,5^). Particularly, in the functional connectivity (FC) approach, pair-wise synchronisations across brain regions successfully revealed diverse aspects of brain network alterations (e.g. ageing^6^ and developmental processes^7^), including pathological conditions (e.g. schizophrenia^8^ and Alzheimer’s disease [AD]^9^) (reviewed in^10–12^). Furthermore, recent findings on brain networks have shown that FC is not static but dynamically changes over time, even without external stimuli, which is called dynamic functional connectivity (dFC)^12–16^ (reviewed in^17^). This dynamic property reflects the ability of brain function^18–20^ and characterises both healthy ageing^21,22^ and pathological conditions^23^.

Neural activity involves moment-to-moment dynamics occurring within milliseconds^24–26^ (reviewed in^27,28^). To evaluate these high-frequency components in dFC, a neuroimaging modality with a high temporal resolution, typified as electroencephalography (EEG) or magnetoencephalography, is appropriate^13,29,30^. Despite its temporal significance, a sliding-time-window approach (focusing on the variability in synchronisation between neural activities within a certain time window) has been applied^15,16^ in the conventional analysis of dFC, which deprives us of high temporal resolution of the neuroimaging data. To address this issue, we recently introduced a dynamic phase synchronisation (DPS) approach based on the temporal complexity of the instantaneous phase difference between neural activities. This approach enables the detection of the instantaneous characteristics of dFC involving historical characteristics (or deterministic characteristics) produced by inherent network dynamics^31^. Subsequently, this approach could successfully detect the ageing process in the frontal cortical network^31^. Hence, focusing on the instantaneous phase of neural activity provides additional insights into existing dFC analyses^31^.

In the dFC approach including DPS, complex and multiple network dynamics are captured by pair-wise interactions of neural activities. In contrast, an approach with a microstate, which is an intermittently changing quasi-stable state defined by a spatial power distribution, captures the dynamic state of whole-brain neural activity^32,32,33^ (reviewed in^34^). This microstate reportedly reflects the global integration corresponding to multiple neural interactions among brain regions and dynamic state transition of the whole brain network in cognitive processes^35–39^. Moreover, a large number of studies comparing EEG activity and dFC^15,40–44^, Abreu *et al. * have recently demonstrated that the microstate reflects the global network pattern in dFCs estimated by functional magnetic resonance imaging with precise accuracy^45^, rather than focusing on local EEG activity in individual brain regions. Even though the conventional microstate based on power distribution reflects whole-brain interactions, the application of the microstate approach to instantaneous phase interactions involving moment-to-moment dFC^31^ might lead to a much more progressive approach to detect dynamical whole-brain interactions. As a preliminary study, our recent study reported that the spatial distribution of instantaneous frequency (IF) in EEG signals exhibited region-specific patterns and their temporal transitions, which resemble conventional microstates based on power distribution, that is, identification of brain region-specific leading phase states and their dynamical emergence/transitions^46^.

Impairment in the integration of neural activity leads to various pathological conditions involving cognitive decline (reviewed in^10–12^). Particularly, due to the progress of the ageing society, the global prevalence of AD, which is the most common form of dementia, will increase to 0.6% in 2030 and 1.2% by 2046^47^. Although effective treatments for AD are controversial, recent studies have shown that the early diagnosis of AD and early intervention significantly delay disease evolution^48^. Therefore, the advent of biomarkers that support early diagnosis is expected. AD causes cognitive impairment due to the loss of multiple neural interactions induced by progressive neuronal death, neurofibrillary tangles, and senile plaques in widespread brain regions^49–51^. The use of the phase synchronisation approach has revealed that the progression of AD leads to alterations in frequency-band-specific global FC^26,52^. Moreover, studies on whole-brain network dynamics captured by dFC analysis using a sliding-time-window^53–55^ and microstate analysis based on power spatial distribution^56–58^ reported alterations in network dynamics in AD.

In this context, in addition to the DPS approach, where the instantaneous phase of neural activity is applied to the evaluation of dFC^31^, we hypothesised that introducing a microstate based on the instantaneous phase reflecting the moment-to-moment dynamical characteristics of whole-brain network activity might provide another dimension of understanding to the network alternation in AD. To validate this hypothesis, first, based on the findings of the region-specific IF distribution^46^, we developed a novel microstate analysis with the dynamics of deviation for spatial IF distribution in the whole brain and applied it to AD EEG signals. Subsequently, we validated whether the time evolution of whole-brain IF reflected the inherent dynamic process in the neural network. Finally, we validated the effectiveness of the IF microstate in identifying alterations in network dynamics due to AD pathology.

## Results

### Analysis for the dynamics of the IF in EEG signals

**Figure 1:**
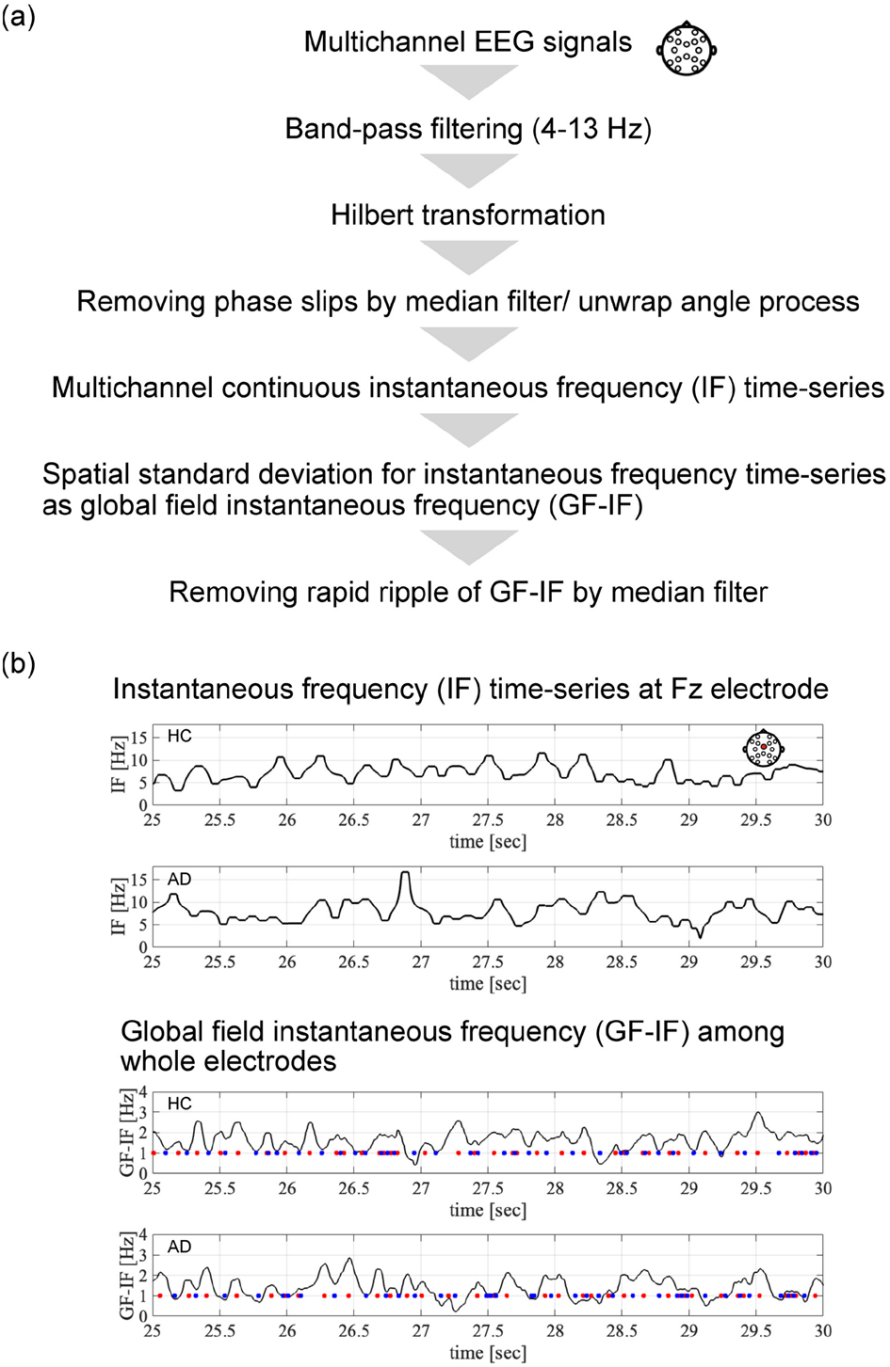
(**a**) Estimation process for the continuous instantaneous frequency (IF) time-series of electroencephalography (EEG) signals and global field instantaneous frequency (GF-IF) based on IFs among whole electrodes. (**b**) Typical examples of time-series of the IF at the Fz electrode (upper part) and GF-IFs (lower part) in for healthy control (HC) and Alzheimer’s disease (AD) cases. Red and blue dots exhibit the local maximum and minimum of GF-IFs. Here, the duration of 25–30 s is shown within the whole evaluation period.

First, we provide an overview of the estimation method for the IF and temporal behaviour of the spatial deviation, as shown in Fig. 1a. In this method, multichannel EEG signals were processed by band-pass filtering to extract oscillations in the frequency regions involving theta and alpha [4 : 13] Hz, which are the dominant oscillations in the resting eye-closed EEG signals in both healthy control (HCs) and patients with AD. In microstate analysis involving conventional methods, the frequency band is used over a wider range than FC^34^. Subsequently, by Hilbert transformation, the multichannel time series of the IF was derived through processing to remove phase slips using a median filter and an unwrapping angle. Typical examples of IF time series at the Fz electrode are shown at the top of Fig. 1b. The IFs at all electrodes fluctuated around the frequency range of band-pass filtering (see the movies for temporal evolution of the IF at all electrodes in the Supplementary Information). From these results, owing to the slowing wave of AD, the temporal and spatial averages of the IF in the AD group were significantly low ($t=-2.8016, p=0.0099$) (group-average of HC cases: 8.58 Hz, group-average of AD cases: 7.82 Hz); the spatial patterns of the IFs were transient, intermittent in both groups. To capture the characteristics of this evolution, the time series of the standard deviation of the IFs among all electrodes was derived, which is called the global field instantaneous frequency (GF-IF) in this study (see the bottom part of Fig. 1a). Here, the high-frequency ripple behaviour in the GF-IF was eliminated using the median-filtering process. As shown at the bottom of Fig. 1b, the GF-IF exhibits the fluctuated oscillations in both HCs and patients with AD. The temporal averages of the GF-IF are not different between HCs and patients with AD [$t=-1.151,p=0.258$ (group average of HC cases: 1.486 Hz, group-average of AD cases: 1.425 Hz)].

**Figure 2:**
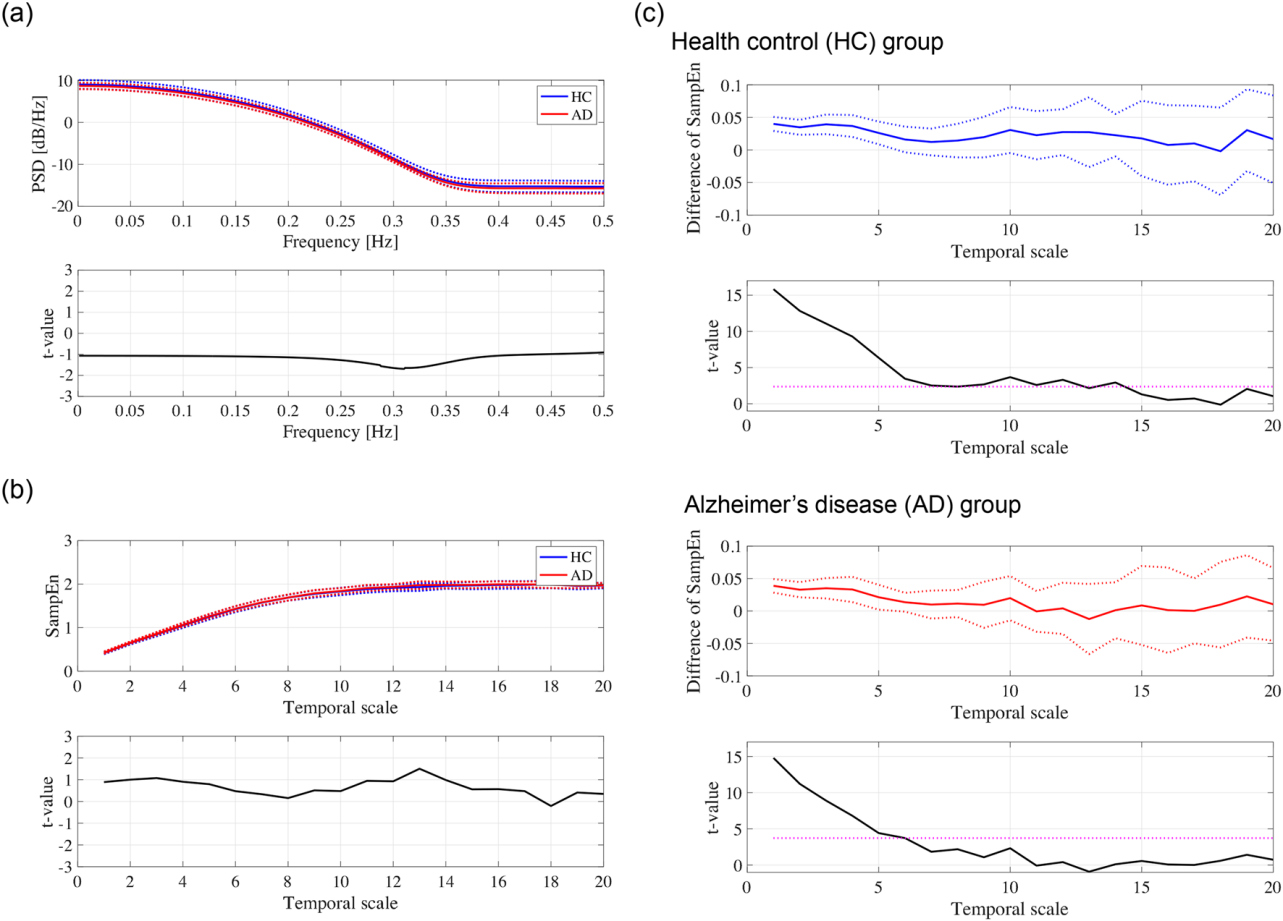
Dynamical characteristics of the GF-IF. (**a**) Average of power spectrum density (PSD) of the GF-IF in the HC and AD groups (dotted lines exhibit the standard deviations) (upper part). *t*-value between the HC and AD groups (lower part). Positive (negative) values correspond to a larger (smaller) PSD for the AD group than for the HC group. No significant difference was confirmed. (**b**) Average of sample entropy (SampEn) dependency on the temporal scale in the HC and AD groups (dotted lines exhibit the standard deviations) (upper part). *t*-value between the HC and AD groups (lower part). Positive (negative) values correspond to a larger (smaller) SampEn for the AD group than for the HC group. (**c**) Difference in SampEns between the original GF-IF time-series and iterated amplitude adjusted Fourier transform (IAAFT) surrogates [first (HC) and third (AD) columns] and their paired-*t* value [second (HC) and fourth (AD) columns]. The larger *t*-values than magenta dotted line satisfy with $q<0.05$. The temporal scale components of the temporal patterns of the GF-IF [$\lesssim 6$ ($\lesssim 0.03$ s)] possess the deterministic characteristics.

Second, the frequency characteristics of temporal behaviour in the GF-IF were evaluated. The result of the power spectrum density (PSD) of the GF-IF shows that the frequency component of the GF-IF is distributed in the frequency region $\lesssim 0.35$ [Hz], and there is no difference in PSD between HCs and patients with AD(see Fig. 2a). The dynamic patterns of the GF-IF were analysed using multiscale entropy (MSE) analysis^59^. In the MSE analysis, the time series of the GF-IF were coarse-grained at each temporal scale; these complexities were quantified by sample entropy (SampEn). The MSE profiles of the GF-IF in the HC and AD groups are shown in Fig. 2b. SampEn exhibited a monotonic increase as a function of temporal scale in both the HC and AD cases; these profiles showed no difference between them. Moreover, the deterministic properties of these patterns were evaluated by comparing the MSE profile of the GF-IF time-series and that of the corresponding iterated amplitude-adjusted Fourier transform (IAAFT) surrogates^60^. In Fig. 2c, the differences in SampEn between the original GF-IF and the average of ten IAAFT surrogates produced from different random seeds and paired-*t* values as a function of the temporal scale were represented. In both HC and AD cases, the values of SampEn in the IAAFT surrogates were significantly higher than those in the original GF-IF time-series on the temporal scale [$\lesssim 6$ ($\lesssim$ 0.03 s)]. Thus, the temporal patterns of the GF-IF involve deterministic characteristics produced by the inherent dynamic process of the neural network, that is, the previous state of the neural network reflecting the GF-IF determines the next state, although the GF-IF cannot capture the pathology of AD.

### Analysis for microstates based on the IF

**Figure 3:**
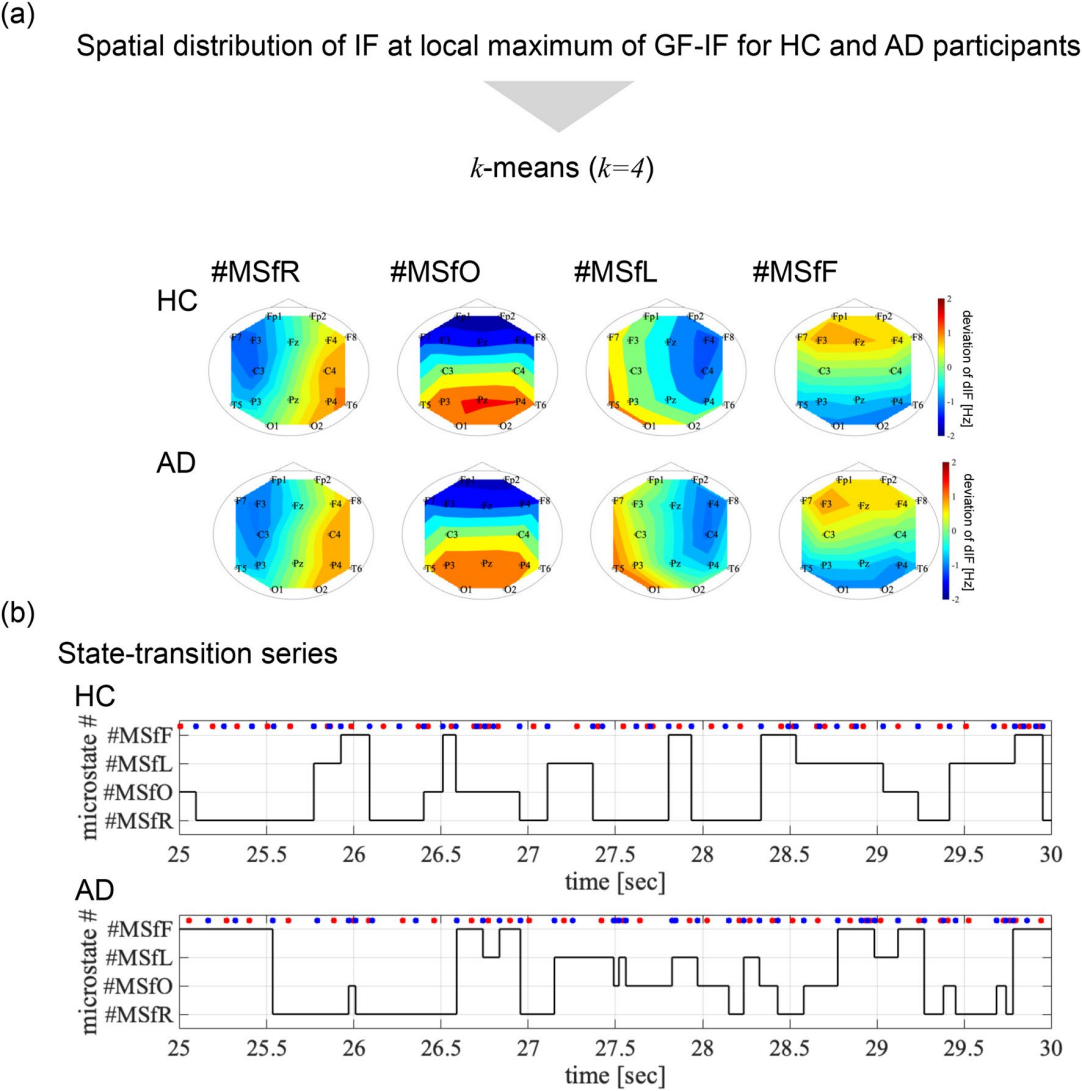
(**a**) Classified spatial distributions of the IFs. The average of the IFs within each classified state, that is, the right-hemispheric, occipital, left-hemispheric, and frontal leading phase microstates were identified in both the HC and AD groups. (**b**) State transitions of typical HCs and participants with AD. Red and blue dots exhibit the local maximum and minimum of the GF-IFs. We defined that the state transition was at the local minimum of the GF-IF, that is, diminishing localisation of the IFs. The intermittent transitions of the IF microstate were confirmed.

Regarding the behaviours of the GF-IF, which reflects the interactions among whole-brain activities, the maximised GF-IF can be interpreted as the appearance of a signal source that corresponds to the fastest neural activity (the highest IF value) among the regions. To capture the characteristics of the intermittent transition of the source location and their alternation owing to the pathology of AD, we classified the IFs at the maximised GF-IF using the *k*-means method (cluster size is set to $k=4$) (see the flow of classification in Fig. 3a); subsequently, the temporal transition of classified states called as the IF microstate was evaluated.

The classified spatial distributions of the IFs, which were temporal-averaged within each classified state, are shown in Fig. 3a. Right-hemispheric, occipital, left-hemispheric, and frontal leading phase microstates were identified in both the HC and AD groups. Under the definition where the state transients at the local minimum of the GF-IF, that is, diminishing the local specification of the IFs, the state transitions of typical participants in the HC and AD groups are shown in Fig. 3b. Intermittent transitions of the IF microstates were confirmed.

**Figure 4:**
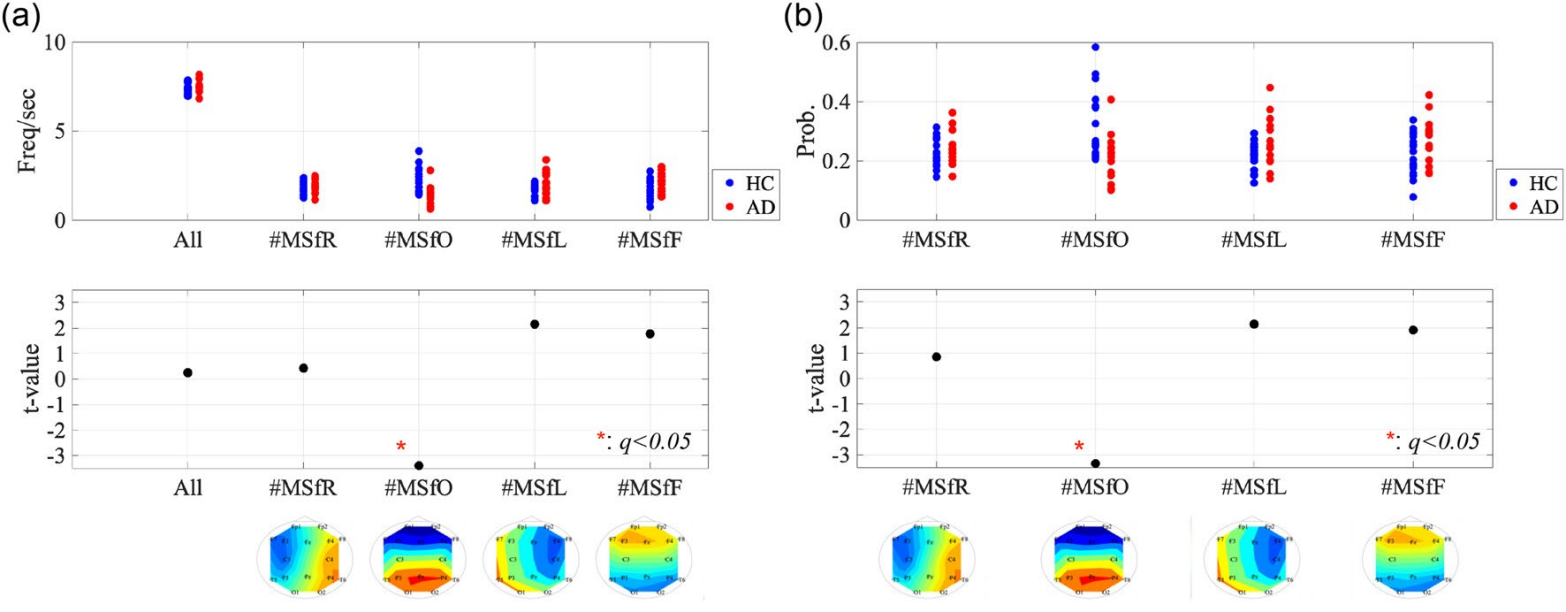
(**a**) Emergence frequency of each microstate per second (all: frequency of all IF microstates, #MSfR: right-hemispheric, #MSfO: occipital, #MSfL: left-hemispheric, and #MSfF frontal leading phase microstates). (**b**) Rate of occurrence duration for each IF microstate. The significant decreasing emergence frequency and rate of occurrence duration for the occipital leading phase ($t=-3.400$ ($p=0.0018$) and $t=-3.351$ ($p=0.0020$), respectively, satisfying with $q<0.05$) were confirmed in the AD group.

**Figure 5:**
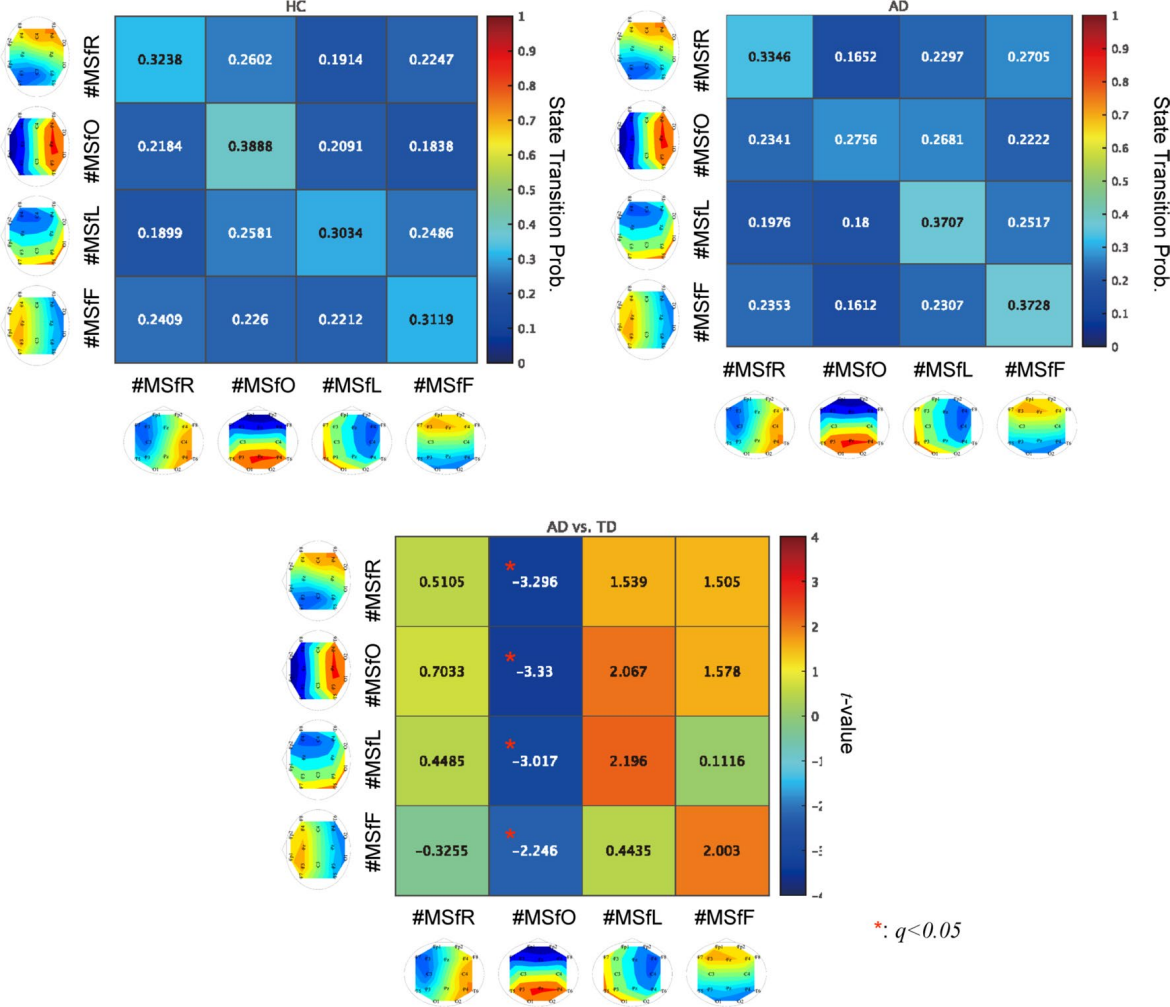
Mean state transition probability from the source state (vertical axis) to the destination state (horizontal axis) in the HC and AD groups (upper part). The positive (negative) *t*-value corresponds to a high (low) probability of participants with AD than HCs (lower part). The result showed the significant decreasing the state transition probabilities from the frontal, left-hemispheric, and occipital leading states to occipital leading state in AD cases, satisfying with $q<0.05$.

To evaluate the temporal characteristics of the transition, Fig. 4 shows the emergence frequency per second (a) and rate of occurrence duration for each IF microstate (b). Consequently, a significant decrease in the emergence frequency and rate of occurrence duration for the occipital leading phase ($t=-3.400$ ($p=0.0018$) and $t=-3.351$ ($p=0.0020$), respectively, satisfying $q<0.05$) was confirmed in the AD group. Furthermore, to evaluate the temporal transition probability among these IF microstates, we analysed the mean state transition probability across all four microstates in the HC and AD groups and differences between the groups (Fig. 5). Consequently, a significant decrease was observed in the transition probability from the frontal, left-hemispheric, and occipital leading states to the occipital leading state in AD, satisfying $q<0.05$.

**Figure 6:**
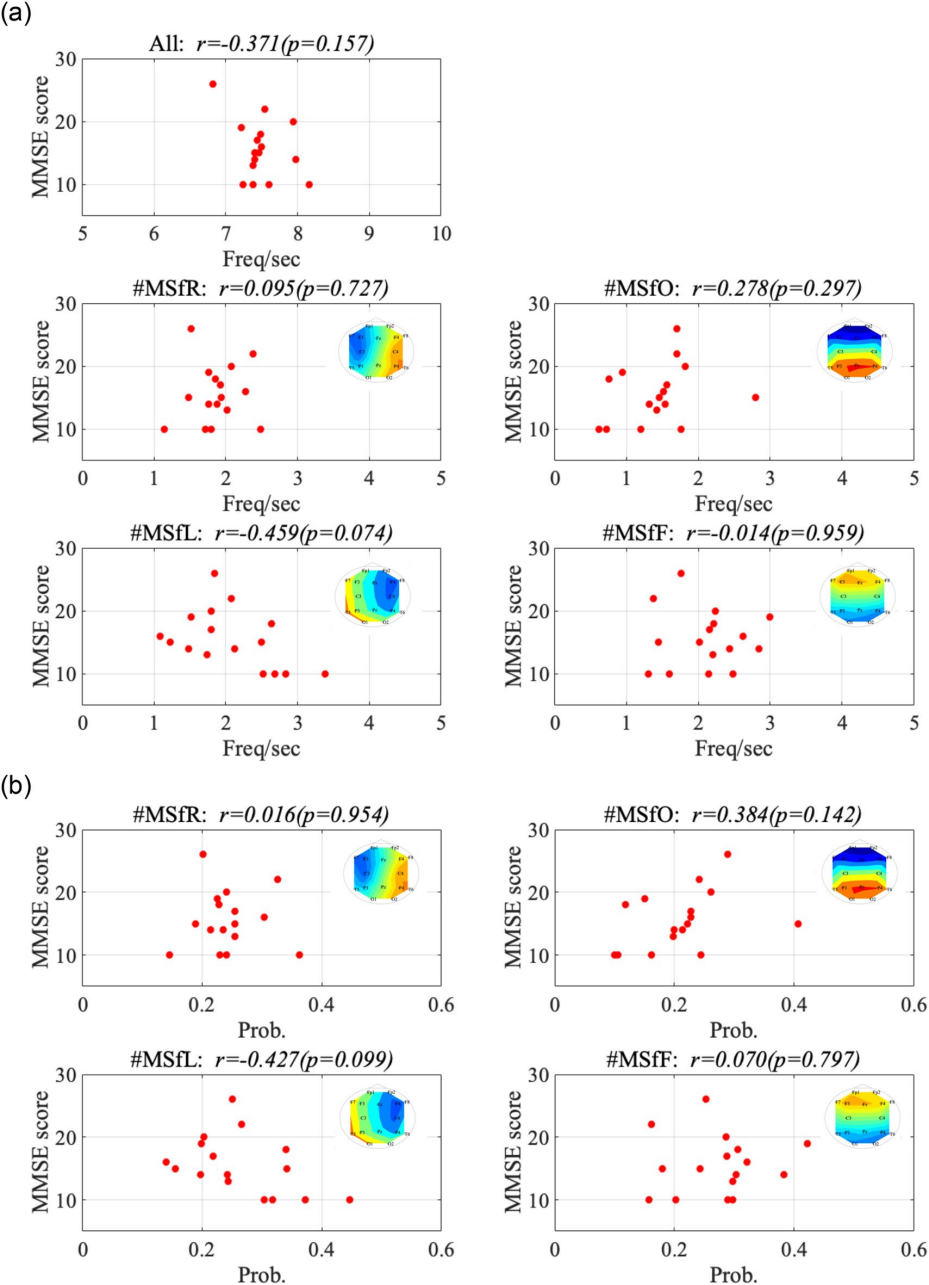
(**a**) Scatter plots between the emergence frequency (corresponding to Fig. 4a) and Mini-Mental State Examination (MMSE) scores and their correlation coefficient *r*. (**b**) Scatter plots between the rate of occurrence duration (corresponding to Fig. 4b) and MMSE scores and their *r*. No correlations were observed in both cases.

**Figure 7:**
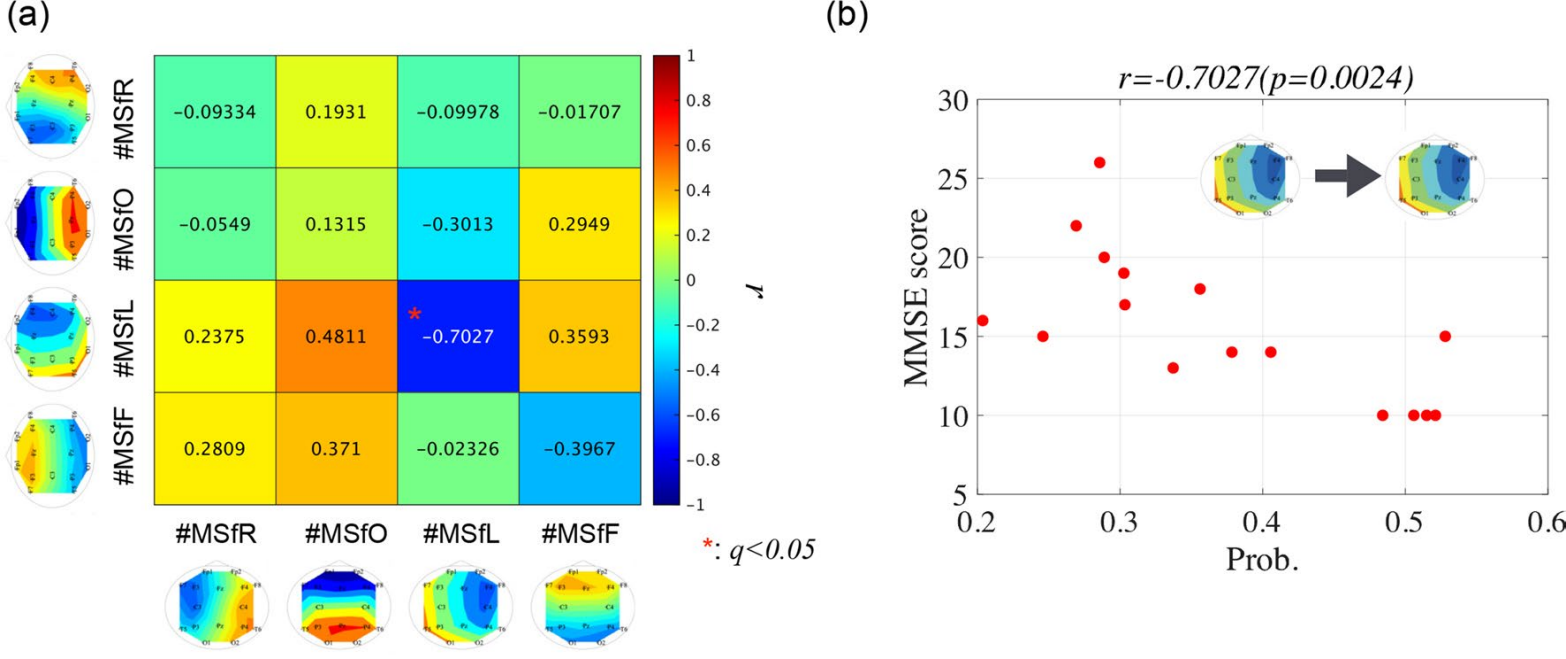
(**a**) Correlation coefficient *r* between the state transition probability and MMSE scores. (**b**) The associated scatter plot with a significantly large correlation coefficient *r* ($q<0.05$). The result showed that a significantly large negative correlation for the transition probability from the left-hemispheric leading state to the left-hemispheric state.

Furthermore, we investigated the relationship between IF microstates and cognitive function in AD. Figure 6 shows the scatter plots of the emergence frequency (corresponding to Fig. 4a) and mini-mental state examination (MMSE) scores and their Pearson’s correlation coefficient *r* and scatter plots between the rate of occurrence duration (corresponding to Fig.4b) and MMSE scores and their *r*. The results showed that correlations with the statistical criterion $q<0.05$ were not observed in either case. In Fig. 7, the Pearson’s correlation coefficients *r* between the state transition probability and MMSE scores (a) and scatter plots with the significant large correlation coefficient *r* ($q<0.05$) (b) are shown. The results show a significantly large negative correlation between the transition probability from the left-hemispheric leading state to the left-hemispheric state. Thus, it can be interpreted that over maintenance of the left-hemispheric leading state and difficulty in transitioning from the left-hemispheric leading state might induce cognitive decline.

## Discussion

In this study, we aimed to investigate the inherent instantaneous spatiotemporal neural dynamics of the human brain and their alterations in patients with AD. To this end, a microstate approach was applied to the whole-brain IF distribution of EEG signals. Our results indicate that the temporal fluctuation of the whole-brain IF, which is captured by the GF-IF, reflects the internal dynamical process of neural networks; moreover, in the microstates based on this whole-brain IF, the emergence frequency/rate of occurrence duration of occipital leading state in patients with AD decreased in comparison with that in HCs. Regarding the state transition among the IF microstates, the state transition probabilities of the occipital-to-frontal, occipital-to-left-hemispheric, and occipital-to-occipital leading states patients with AD decreased compared with those in HCs, that is, participants with AD were harder to transition to the occipital leading state and easier to transition from the occipital leading state. The state transition probabilities of the left-hemispheric-to-left-hemispheric leading states exhibited a significantly large negative correlation with the MMSE. In other words, difficulty in transitioning from a left-hemispheric leading state is correlated with cognitive decline.

First, we must consider the reason why the temporal evolution of the spatial deviation of the IFs, that is, the GF-IFs, reflects the inherent dynamic process. Many studies using the phase synchronisation approach for FC have revealed that the instantaneous phase component reflects the interaction of inter-regional neural activity^6–12^. Moreover, in addition to the evaluation of static phase synchronisation within a certain time window in the FC evaluation, the moment-to-moment interaction, which appears in the instantaneous phase difference captured by the DPS approach, exhibits deterministic characteristics produced by internal network dynamics^31^. Therefore, the whole-brain assembly of these phase components as the GF-IF inherited this deterministic characteristic (Fig. 2). However, in the GF-IF, the multi-dimensional IF behaviours, involving whole brain interactions, are degenerated to one-dimensional behaviours without spatial region specificity; consequently, the GF-IF cannot capture the pathology of AD for the relatively small and heterogeneous clinical dataset used in this study^25,26^.

In contrast to the GF-IF, the IF microstate as a whole-brain IF distribution can reflect the regional specificity of IF interactions, and we must discuss the difficulties in maintaining occipital leading states and transition to occipital leading states related to AD pathological conditions. The pathological progression of AD leads to the impairment of the posterior cingulate gyrus, which plays an important role in the integration and transmission processes as a hub for brain networks (reviewed in^61–63^). Assuming that the leading-phase state corresponds to the transmission process^64^, the hub function in AD progression with cognitive decline^65–67^ may induce difficulties in the occipital leading state (see Figs. 4 and 5).

Further, we discuss the correlation of the difficulty in transitioning from the left-hemispheric leading phase state with cognitive decline in the AD group. Almost all evaluated items for cognitive functions measured by the MMSE relate to language and memory functions^68^. The brain regions involved in verbal memory functions are located in the left hemisphere of the brain, especially in the left-sided hippocampus^69,70^; progression in AD leads to hippocampal shrinkage^71–73^ and dysfunction in the integration of verbal memories and semantic concepts^74–76^. Considering these findings, the low frequency of transience from the left-hemispheric leading phase state might reflect diminishing interactions starting from the left hemisphere as the source of verbal memory function, that is, diminishing dFC, in AD with a reduced MMSE score. However, for a more detailed verification of this finding, a comparison between the IF microstate and exhaustive cognitive function tests in a larger dataset is needed.

Additionally, we must consider the comparison with comparison with the microstate approach based on signal amplitude, similar to the conventional microstate approach. In “Supplementary Note 4”, the microstates based on instantaneous amplitude (IA) were evaluated under the condition with the same frequency band (4–13 Hz) and clustering method. The result showed similar spatial patterns (left-hemispheric, occipital, right-hemispheric, and frontal activate micro-state). In contrast to the micro-state based on IF, the right-hemispheric activate state based on IA in the AD group emerges with a significantly higher frequency. Therefore, IF might capture a different aspect of network alterations in the pathology of AD. However, to reveal a more detailed mechanism to produce each IF microstate and its alternation, magnetoencephalography and complementary cortical source localization techniques are needed, as the EEG signals used on the scalp cannot precisely identify the source of neural activity inducing the IF microstate. Furthermore, recent studies are focusing on the relationship between the microstate and dFC^77,78^. The other type of microstate approach based on dFC, which is defined as dynamics of FC among all the electrodes, instead of each phase component of EEG signals used in this study, has been proposed^79,80^. Evaluation from the viewpoint of the multiple microstate approach may introduce a novel and comprehensive perspective regarding the complex dynamics of the brain.

Finally, the limitations of this study should be considered. First, we set *k* cluster numbers to estimate the IF microstate based on an instantaneous distribution to $k=4$, which is widely utilised in the conventional power-based microstate approach^32,32,33^. However, this cluster number might be optimised for the IF microstate based on the instantaneous distribution. Moreover, according to the tendency of silhouette value^81^ (see “Supplementary Note 3”), the use of $k=4$, corresponding to the setting for the conventional EEG microstates approach, is deemed necessary for the analysis of neuroimaging with high spatial resolution, typified by high-density EEG. Second, the effect of volume conduction can lead to spurious synchronization in the estimation of FC. Therefore, metrics of functional connectivity often incorporate measures to address this effect^82^. However, it is unclear whether volume conduction can affect to the state based on the phase components of EEG signals across all electrodes in our proposed IF microstate approach, as well as pair-wise synchronization. Therefore, this point needs clarification; we must develop a method to address this influence if volume conduction significantly affects the IF microstate. Third, this study dealt with participants with mild or higher severity of AD; therefore, evaluation of participants with mild cognitive impairment is needed to apply the IF microstate approach to the early diagnosis of AD. Fourth, alterations in the network dynamics involving dFC appear in many types of psychiatric disorders^83,84^. Therefore, alterations in dynamic states based on the IF distribution might emerge in these disorders, and such disease-specific characteristics must be revealed. In future studies, we plan to address these issues.

## Conclusions

In this study, we introduced a new microstate approach based on whole-brain IF distributions. This approach can be used to detect alterations in the network dynamics that reflect AD pathology and cognitive decline. Although some limitations remain, this approach might become an effective approach for revealing the brain functions produced by whole-brain interactions as emerging phenomena and their alternations.

## Material and methods

### Participants

We included 16 participants with AD who satisfied the National Institute of Neurological and Communicative Disorders and Stroke-Alzheimer’s Disease and Related Disorders Association criteria and were in a state before the onset of primary dementia according to the Diagnostic and Statistical Manual of Mental Disorders (fourth edition) criteria, and sex- and age-matched healthy older participants who were non-smokers and were not on medication^25,26,85^. In the HC group, participants with medical or neurological conditions involving epilepsy or head trauma in the past and with a history of alcohol or drug dependency were excluded. In patients with AD, medications affecting the central nervous system were not administered, and the Functional Assessment Staging Test (FAST) and MMSE^68^ were conducted. There were three participants with mild AD (FAST 3), seven with moderate AD (FAST 4), and six with severe dementia (FAST 5). The MMSE scores were distributed in the range of 10–26 (average: 15.56). Detailed information about the participants is provided in Table 1. Informed consent was obtained from all participants before the study. The experimental protocol of this study was approved by the Ethics Committee of Kanazawa University and conducted in accordance with the Declaration of Helsinki.

**Table 1:** Physical characteristics of healthy older controls (HCs) and patients with Alzheimer’s disease (AD).

	HC participants	AD participants	*p* values
Male/female	7/11	5/11	0.72
Age (year)	59.3 (5.3, 55–66)	57.5 (4.7, 43–64)	0.31
MMSE score	NA	15.5 (4.7, 10–26)	NA

Here, the MMSE indicates the Mini-Mental State Examination.

### EEG recording

To conduct EEG recording, 16 electrodes located at Fp1, Fp2, F3, F4, C3, C4, P3, P4, O1, O2, F7, F8, Fz, Pz, T5, and T6 according to the international 10–20 system of electrode placement were used. EEG signals were measured using a binaural connection as a reference. During the EEG measurements, the participants sat in an electrically shielded and soundproof recording room, and the room illuminance was controlled. The EEG-4518 apparatus (Nihon Kohden, Tokyo, Japan) was used for EEG measurements. The EEG signal was recorded at a sampling frequency of 200 Hz and a bandpass filter of 1.5–60 Hz. The electrode/skin conductance impedance was carefully controlled at each electrode to be less than $5 \text {k}\Omega$. Eye movements were traced using bipolar electrocardiography. Each participant’s EEG signal was measured for 10–15 min in a resting state with eyes closed. In the recorded EEG signals, an artifact-free continuous 60s (12, 000 data points) epoch was selected for each participant.

### Microstate based on spatial patterns of the IF

In this study, the states of brain activity were defined using the instantaneous phase dynamics of EEG signals in 16 electrodes. The instantaneous phase dynamics were estimated using the following process (an overview is shown in Fig. 1). The time series of the multichannel EEG signals were band-pass filtered for the frequency range [4 : 13] Hz, which was set to involve the dominant frequency component of EEG activity in HCs and participants with AD with slowing waves (see for the profile of power spectrum in “Supplementary Note 1”)^86^. The same tendency of spatial patterns of microstate was confirmed using the wider frequency band used in conventional microstate analyses [2 : 20] Hz^34^(see “Supplementary Note 1”). The first and last 5-s periods (1000 data points) in each bandpass-filtered epoch were removed to avoid distortions produced by the bandpass filtering process. The wrapped instantaneous phase $\theta (t)$ ($-\pi \le \theta \le \pi$) was estimated through the Hilbert transformation. This instantaneous phase involves phase noise that causes a significantly large deviation from the instantaneous frequency range [4 : 13] Hz, which is known as a phase slip^87^. Therefore, a median-filtering process with a window length (0.1 s) for the IF was applied. This estimation method was used in the DPS approach in a previous study^31^. The instantaneous amplitude and phase, which were derived from the Hilbert transformation, correspond to the envelope components and higher-frequency oscillations. The conventional microstate approach focuses mainly on the whole-brain distribution of the amplitude corresponding to the instantaneous amplitude^32,33^.

In the state-estimation process based on a continuous instantaneous frequency time series $I\!F_i (t)$ (*i* the electrode location) (its overview shown in Fig.1b), the deviation of $I\!F_i (t)$ from the average $I\!F_i (t)$ among all electrodes:

1 $$\begin{aligned} dI\!F_i(t)=I\!F_i (t)-\overline{I\!F (t)}, \end{aligned}$$

was used ($\overline{I\!F (t)}$ is averaged $I\!F_i (t)$ among all electrodes). $dI\!F_i(t)$ represents the degree of leading (positive value) or delaying (negative value) of the phase component of neural activity in comparison with whole brain regions. The standard deviation of $dI\!F_i(t)$ among all the electrodes is defined as the GF-IF. Here, the high-frequency ripple behaviour in GF-IF was removed by the median-filtering process with a window-length (0.025 s). Based on the time series $dI\!F_i(t)$ at the local maximum of the GF-IF in both the HC and AD groups, the centres of *k* clusters were determined by the *k*-means algorithm, where the Euclidean distance was used to denote the distance in the space of $dI\!F_i(t)$. These centres were estimated for overall participants in both groups (regarding the validity of the length of time-series and the cluster size *k*, these evaluations were shown in “Supplementary Note 2” and “Supplementary Note 3”, respectively). Each cluster corresponds to a dynamic state based on 16 values of $dI\!F_i(t)$. The appearance of state transients was defined as the local minimum of the GF-IF, that is, diminishing the local specification of the IFs. Using *k* clusters, the dynamic transitions among *k* states in both the HC and AD groups were obtained. These transitions were assumed to reflect the moment-to-moment dFC in the whole-brain network. In this study, according to the standard cluster size in the conventional microstate^35–39^, a cluster size of $k=4$.

### Evaluation indexes for the IF microstate

#### Power spectrum analysis

To evaluate the power spectrum of the behaviours of the GF-IF, we used the PSD in dB/Hz using Welch’s method with a Hanning window function width of 5.0 s. The frequency and bin were set to [0.001 : 1] Hz and 0.001 Hz, respectively. The PSD was estimated using the signal-processing toolbox in MATLAB. To assess the difference in the PSD between the AD and HC groups, a *t*-test was conducted. Benjamini-Hochberg false discovery rate (FDR) correction was applied to the *t*-score for multiple comparisons ($q<0.05$) (1000 *p* values).

#### Multi-scale entropy analysis

MSE analysis was utilised to assess the temporal scale dependence of the GF-IF time-series complexity^59^. The time-series sample entropy of random Z-score variables {$x_1,x_2,...,x_N$} is given by

2 $$\begin{aligned} h(r,m) = -\log \frac{C_{m+1}(r)}{C_m(r)}. \end{aligned}$$

    Here, $C_m(r)$ is the probability of $\Vert \textbf{x}^m_i-\textbf{x}^m_j\Vert < r$ $(i\ne j, i,j = 1,2,...)$ among all pairs of *i* and *j*. $\textbf{x}^m_i$ indicates an *m*-dimensional vector $\textbf{x}^m_i = \{x_i,x_{i+1},...,x_{i+m-1}\}$. In the MSE analysis, $\{x_1,x_2,...,x_N\}$ was calculated using Eq. (3) for the coarse-grained time series $y_j$:

3 $$\begin{aligned} x_j = \frac{1}{\tau } \sum _{i=(j-1)\tau +1}^{j_\tau } y_i (1 \le j \le \frac{N}{\tau }). \end{aligned}$$

    Here, $\{y_1,y_2,...,y_{N}\}$ represents the observed signals. $\tau (\tau = 1,2,...)$ represents a temporal scale. In this study, we set $m = 2$ and $r = 0.2$^59^ and MSE analysis was performed using the Physio Toolkit toolbox in MATLAB (http://physionet.incor.usp.br/physiotools/sampen/). To assess the difference in SampEn between the AD and HC groups at each temporal scale, a *t*-test was conducted. FDR correction was applied to the *t*-score for multiple comparisons ($q<0.05$) (20 *p* values).

To investigate whether a nonlinear dynamic process was involved in the temporal behaviour of the GF-IF in both the HC and AD groups, we used IAAFT surrogate data analysis^60^ with an iteration number of 100. Ten IAAFT surrogate datasets were generated using different random seeds for each original GF-IF. The SampEn values were averaged and compared with the values from the original GF-IF. To assess the difference between SampEn of the original GF-IF and that of the surrogate datasets, a paired *t*-test was conducted. FDR correction was applied to the *t*-score for multiple comparisons ($q<0.05$) (20 *p* values).

#### IF microstate analysis

To capture the temporal behaviours of each microstate, we used the emergence frequency, rate of occurrence duration, and sustaining duration of each IF microstate. The emergence frequency was defined as the frequency of the local maximum GF-IF per second. This frequency was counted in the appearances of all the IF microstates and each IF microstate. The rate of occurrence for each IF microstate is given by [summation of the duration between the local minimum of the GF-IF for each IF microstate]/[evaluation duration].

To evaluate the state transitions among IF microstates, we used the probability of state transitions among IF microstates. State transition is defined as the transition probability of the IF microstate from *l*th local maximum GF-IF to $l+1$th local maximum GF-IF.

### Statistical analyses for microstates

To investigate the characteristics of the appearance of the microstate, we assessed the emergence frequency per second and rate of occurrence for each state. For comparisons between the AD and HC groups, *t*-tests were used. FDR correction was applied to the *t*-score for multiple comparisons of the emergence probability ($q<0.05$) (5 *p* values for the emergence frequency and 4 *p* values for the rate of occurrence). Additionally, *t* tests were used to assess the differences in state transition probability between the AD and HC groups, *t*-tests were used. FDR correction was applied to the *t*-score for multiple comparisons of the transition probability ($q<0.05$) (16 *p* values: $k\times k$ state transitions).

Moreover, to reveal the relationship between the emergence frequency, rate of occurrence duration, and transition probability with cognitive functions in the AD group, we evaluated the Pearson’s correlation coefficients *r* with MMSE scores. The statistical criterion for significance was set to $q<0.05$ by adjusting FDR correction.

### Supplementary Information

Supplementary Information.

Supplementary Video 1.

Supplementary Video 2.

## Data Availability

The datasets generated for this study will not be made publicly available, because informed patient consent will not include a declaration regarding the public availability of the clinical data. Requests to access the datasets were made by the corresponding author.
