# Supplementary material for: Atypical instantaneous spatio-temporal patterns of neural dynamics in Alzheimer’s disease
Source: Sci Rep. 2024 Jan 2;14:88. doi: 10.1038/s41598-023-50265-3 (PMC10761722; doi:10.1038/s41598-023-50265-3)
Supplement: Supplementary file 1 — Supplementary Information. [file 41598_2023_50265_MOESM1_ESM.pdf]

Supplementary material for: Atypical Instantaneous Spatio-temporal Patterns of Neural Dynamics in Alzheimer's Disease

Sou Nobukawa[1,2,3*], Takashi Ikeda[4], Mitsuru Kikuchi[4,5], Tetsuya Takahashi[4,6,7]

[1*]Department of Computer Science, Chiba Institute of Technology, 2-17-1 Tsudanuma, Narashino, 275-0016, Chiba, Japan.
[2]Research Center for Mathematical Engineering, Chiba Institute of Technology, 2-17-1 Tsudanuma, Narashino, 275-0016, Chiba, Japan.
[3]Department of Preventive Intervention of Psychiatric Disorders, National Institute of Mental Health National Center of Neurology and Psychiatry, 4-1-1 Ogawa-Higashi, Kodaira, 187-8661, Tokyo, Japan.
[4]Research Center for Child Mental Development, Kanazawa University, 13-1 Takaramachi, Kanazawa, 920-8640, Ishikawa, Japan.
[5]Department of Psychiatry and Behavioral Science, Kanazawa University, 13-1 Takaramachi, Kanazawa, 920-8640, Ishikawa, Japan.
[6]Department of Neuropsychiatry, University of Fukui, 23-3 Matsuoka, Yoshida, 910-1193, Fukui, Japan.
[7]Uozu Shinkei Sanatorium, 1784-1 Eguchi, Uozu, 937-0017, Toyama, Japan.

*Corresponding author(s). E-mail(s): nobukawa@cs.it-chiba.ac.jp;
Contributing authors: tikeda@med.kanazawa-u.ac.jp;
mitsuruk@med.kanazawa-u.ac.jp; takahash@u-fukui.ac.jp;

Supplementary Note 1: Power spectrum of EEG signals and microstates based on broadband IF

In this study, we focused on the main component of EEG signals under the eye-closed condition. Under this condition, the alpha-band oscillations emerged dominantly. However, in the participants with Alzheimer's disease (AD), due to the slowing wave, the alpha-band oscillations shifted to theta-band range. To demonstrate these characteristics [1], Supplementary Fig.1 shows the power spectrum density (PSD) spectrum of EEG signals in the healthy control (HC) and AD groups. Here, PSD in dB/Hz used Welch's method with a Hanning window function width of 5.0 s. The frequency and bin were set to [1 : 60] Hz and 1 Hz, respectively. The PSD was estimated using the signal-processing toolbox in MATLAB. To assess the difference in the PSD between the AD and HC groups, a t-test was conducted. Benjamini–Hochberg false discovery rate (FDR) correction was applied to the t-score for multiple comparisons ($q < 0.05$) (960 p values). The results showed that peaks of PSDs distribute at around 4-13 Hz in both groups.

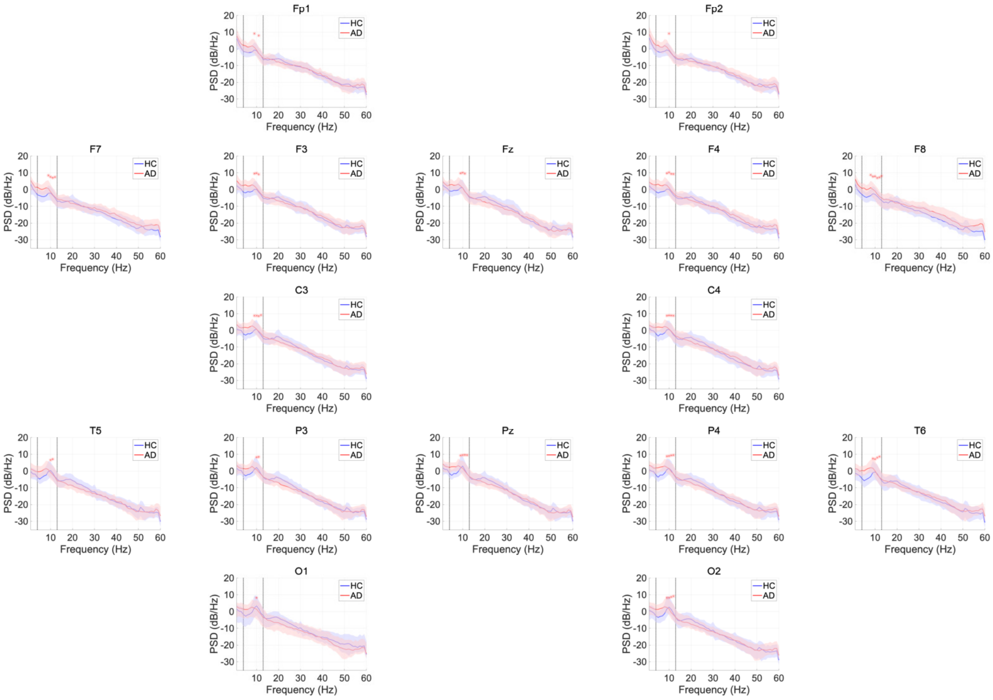

Supplementary Figure 1 Power spectrum density (PSD) spectrum of electroencephalography (EEG) signals in the HC and AD groups. The solid line and shaded area represent the mean and standard deviation of PSD among participants in each group, respectively. Red asterisks indicate significant difference ($q < 0.05$) between healthy control (HC) and Alzheimer's disease (AD) cases. The vertical black dotted lines represent the range of the theta and alpha band: $4 - 13$ Hz used in this study for the estimation of microstates.

In our proposed microstates based on instantaneous frequency (IF), we used a frequency band involving theta (4-8 Hz) and alpha (8-13 Hz), which is narrower than the frequency band of 2-20 Hz used in conventional microstate analyses (reviewed in [2]). In this supplementary material, we evaluated the influence of the width of the frequency band on the estimated microstate. Supplementary Figure 2 shows the estimated spatial patterns of the microstate using a broader range of 2-20 Hz. This result indicates similar spatial patterns-i.e., #MSfR (right hemispheric-leading), #MSfO (occipital-leading), #MSfL (left hemispheric-leading), and #MSfF (frontal-leading) states-to the case with the narrower frequency band (4-13 Hz). Therefore, the estimated microstates exhibit a certain degree of robustness with respect to the setting of frequency bands.

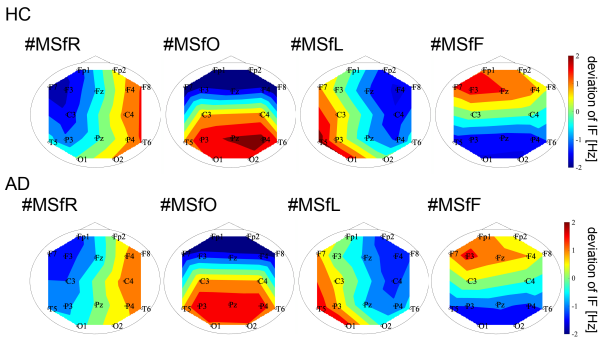

Supplementary Figure 2 Estimated spatial patterns of the microstate using a broader range of 2-20 Hz. #MSfR (right hemispheric-leading), #MSfO (occipital-leading), #MSfL (left hemispheric-leading), and #MSfF (frontal-leading) states, which is similar patterns to the case with the narrower frequency band (4-13 Hz).

Supplementary Note 2: Comparison of centres of cluster between the case with all participants and the case with individual participants

To evaluate the validity of the centres estimated in this study, Supplementary Fig.3 shows the spatial correlation (Spearman's rank correlation coefficient r) between the centres estimated for all participants (corresponding to the centres used in this study) and those estimated for individual participants. The results show that a major part of the centres of individual participants distribute at relatively high correlation ($0.4 \lesssim r \lesssim 1.0$) in both groups. Therefore, we considered the centres of clusters used in this study to be valid for the expression of the states of individual participants in both the HC and AD groups.

Moreover, in addressing the reproducibility of the microstate analysis, Supplementary Fig.4 shows the evaluation of comparing the spatial correlation of cluster centres estimated using the full-length time series of IF for all participants with those estimated using 1/2 and 1/4 length of the time series for individual participants. The results indicate that while a portion of the cluster centres shows a high correlation

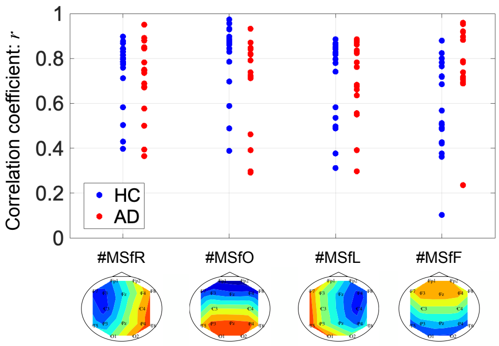

Supplementary Figure 3 Spatial correlation between the centres estimated for all participants (corresponding to the centres used in this study) and those estimated for individual participants in #MSfR, #MSfO, #MSfL, and #MSfF states. #MSfL: left-hemispheric, #MSfO: occipital, #MSfR: right-hemispheric, and #MSfF frontal leading phase microstates

coefficient with the full-length data and all participants, reducing the length of the time series increases variability between and within participants. This suggests that there is variability in the reproducibility both between and within participants. Moving forward, it is important to conduct analyses of this variability using data with a longer follow-up period and larger sample size, considering the implications for clinical diagnosis.

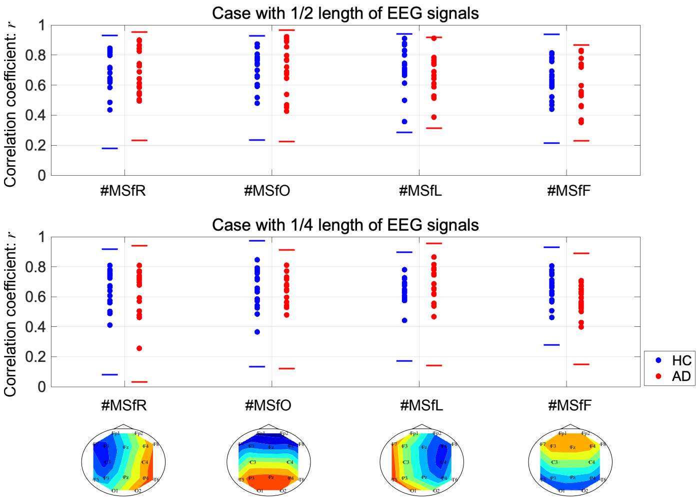

Supplementary Figure 4 Spatial correlation between the centres estimated for full-length time-series of all participants (corresponding to the centres used in this study) and those estimated for using 1/2 and 1/4 length of the time series for individual participants in #MSfR, #MSfO, #MSfL, and #MSfF states. Data points exhibited the mean value among segments within each participant (2 segments in 1/2 length case, 4 segments in 1/4 length case). The horizontal var shows the maximum of minimum values of correlation coefficient among all segments in all participants. #MSfL: left-hemispheric, #MSfO: occipital, #MSfR: right-hemispheric, and #MSfF frontal leading phase microstates

Supplementary Note 3: Evaluation of cluster size for microstates based on IF

To evaluate validity of the number of clusters ($k = 4$), we assessed the silhouette value as the representative index for evaluating the cluster size [3]. The silhouette value ranges from -1 to 1, where approaching 1 indicates high cohesion within each cluster and significant separation among clusters, and a negative value indicates inappropriate clustering. The results for the dependence of the silhouette value on cluster size k (see Fig.5) exhibit a monotonically decreasing tendency while maintaining a positive value. This suggests that, although the $k = 4$ setting may be considered too high, the use of $k = 4$, corresponding to the setting for the conventional EEG microstates approach, is deemed necessary for the analysis of neuroimaging with high spatial resolution, typified by high-density EEG.

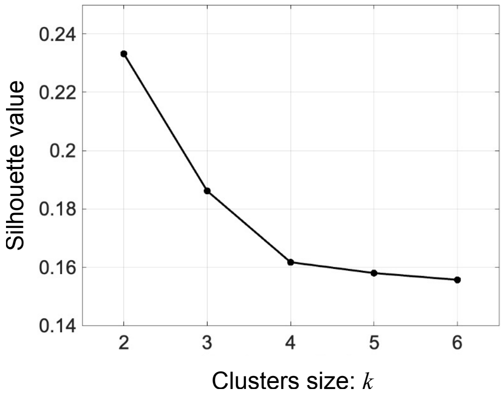

Supplementary Figure 5 Dependence of silhouette value on cluster size k. The centres of clusters were determined by all participants in both the HC and AD groups. The result showed a monotonically decreasing tendency while maintaining a positive value. HC, health control; AD, Alzheimer's disease

Supplementary Note 4: Microstates based on IA

For comparison with the microstate approach based on signal amplitude, similar to the conventional microstate approach, we evaluated the microstates based on instantaneous amplitude (IA) derived by Hilbert transformation, instead of IF, under the condition with the same frequency band (4-13 Hz) and clustering method (see Supplementary Fig.6). Here, ahead of clustering, IA is transformed to the deviation of $IA_i(t)$ from the average $IA_i(t)$ among all electrodes:

$$dIA_i(t) = IA_i(t) - \overline{IA(t)}, \tag{1}$$

was used ($\overline{IA(t)}$ is averaged $IA_i(t)$ among all electrodes). $dIA_i(t)$ represents the degree of activation (positive value) or suppression (negative value) of the amplitude component of neural activity in comparison with whole brain regions. Additionally, $dIA_i(t)$ is

Z-score. The result showed similar spatial patterns, left-hemispheric, occipital, right-hemispheric, and frontal activate microstate, which are denoted as #MSaL, #MSaO, #MSaR, and #MSaF, respectively. In contrast to the micro-state based on IF, the #MSaR state based on IA in the AD group emerges with a significantly higher rate of occurrence duration. Therefore, IF might capture a different aspect of network alterations in the pathology of AD.

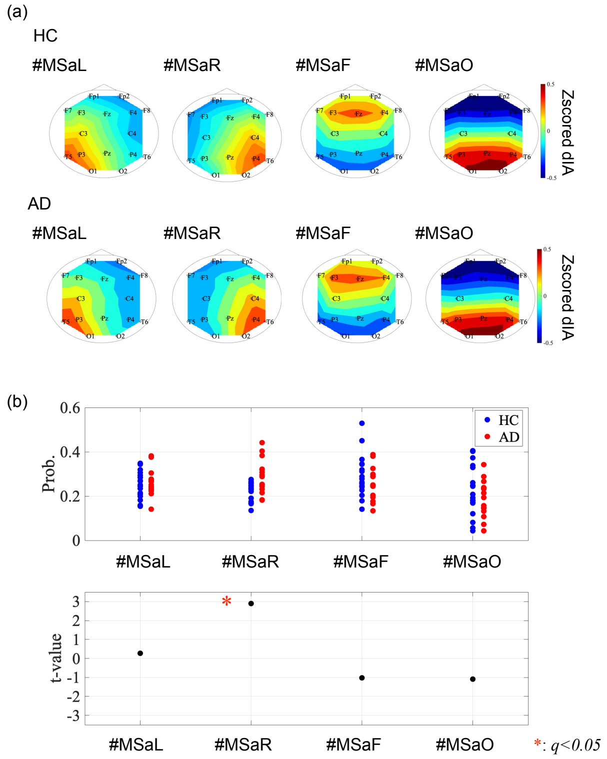

Supplementary Figure 6 (a) Classified spatial distributions of the dIAs. The average of the dIAs within each classified state, that is, left-hemispheric, occipital, right-hemispheric, and frontal activate micro-state, which are denoted as #MSaL, #MSaO, #MSaR, and #MSaF respectively, were identified in both the HC and AD groups. (b) Rate of occurrence duration for each IF microstate. The significant increasing rate of occurrence duration for the #MSaR ($t = 2.901$ ($p = 0.012$), satisfying with criteria of Benjamini–Hochberg false discovery rate (FDR) correction $q < 0.05$) were confirmed in the AD group. HC, health control; AD, Alzheimer's disease; #MSaL, left-hemispheric activate microstate; #MSaO occipital activate microstate; #MSaR, right-hemispheric activate microstate; #MSaF, frontal activate microstate

References

[1] Shinosaki, K., Nishikawa, T., Takeda, M.: Neurobiological basis of behavioral and psychological symptoms in dementia of the Alzheimer type. Psychiatry and Clinical Neurosciences **54**(6), 611–620 (2000)

[2] Michel, C.M., Koenig, T.: EEG microstates as a tool for studying the temporal dynamics of whole-brain neuronal networks: a review. Neuroimage **180**, 577–593 (2018)

[3] Rousseeuw, P.J.: Silhouettes: a graphical aid to the interpretation and validation of cluster analysis. Journal of computational and applied mathematics **20**, 53–65 (1987)
